# Enhancing Geranylgeranyl Pyrophosphate Supply in Engineered Cyanobacteria for Improved Production of Natural Pigments and Terpenoid Precursors

**DOI:** 10.3390/ijms27156680

**Published:** 2026-07-27

**Authors:** Simab Kanwal, Hiran Anjana Ariyawansa, Aphichart Karnchanatat, Tanakarn Monshupanee, Piroonporn Srimongkol

**Affiliations:** 1 High-Value Food from Mushrooms and Bioactive Plants in the Green Economy Value Chain Research Group, The Institute of Biotechnology and Genetic Engineering, Chulalongkorn University, Bangkok 10330, Thailandariyawansa44@ntu.edu.tw (H.A.A.); aphichart.k@chula.ac.th (A.K.); 2 Department of Plant Pathology and Microbiology, National Taiwan University, Taipei 106319, Taiwan; 3 Department of Biochemistry, Faculty of Science, Chulalongkorn University, Bangkok 10330, Thailand; tanakarn.m@chula.ac.th

**Keywords:** *Synechocystis* sp. PCC 6803, MEP pathway, GGPP, metabolic engineering, carbon flux redistribution, isoprenoid biosynthesis

## Abstract

Geranylgeranyl pyrophosphate (GGPP) is a key precursor for carotenoids, chlorophyll derivatives, and numerous high-value isoprenoids used in food, nutraceutical, and pharmaceutical industries. Microbial and photosynthetic production approaches offer promising alternatives to conventional plant extraction and chemical synthesis; however, further optimization and comprehensive sustainability assessments are required for industrial implementation. In this study, a photosynthetic production platform was developed by redirecting carbon flux in *Synechocystis* sp. PCC 6803 to enhance GGPP production via activation of the 2-C-methyl-D-erythritol 4-phosphate (MEP) pathway. Two engineered mutants, ΔP (deficient in poly(3-hydroxybutyrate) synthesis) and ΔGP (deficient in both glycogen and poly(3-hydroxybutyrate) synthesis), were evaluated alongside the wild-type (WT) strain under multiple physiological conditions, including carbon supplementation and UV radiation. Redirecting cellular carbon metabolism significantly enhanced pigment accumulation and overall isoprenoid biosynthesis, with ΔGP strain showing the greatest improvement. Elevated intracellular GGPP levels were observed under all tested conditions, with the highest concentrations obtained under glucose supplementation and UV treatment. The best-performing strain exhibited approximately twelvefold increase in GGPP content relative to the control. These findings demonstrate that rational metabolic engineering of cyanobacteria can markedly improve precursor supply for isoprenoid synthesis, establishing an efficient photosynthetic platform for producing natural pigments, flavor compounds, and other value-added bioproducts.

## 1. Introduction

**Figure 1:**
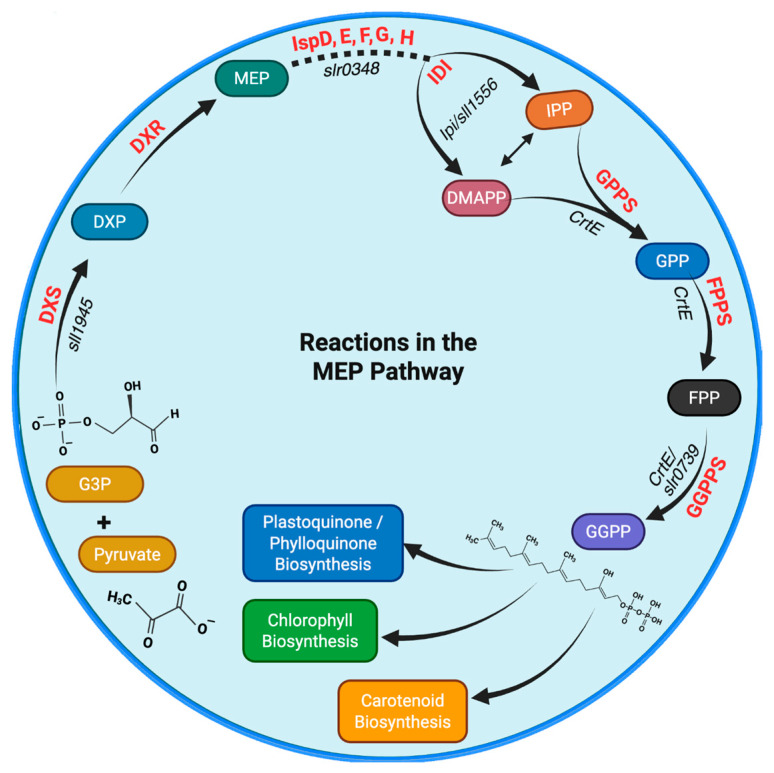
The MEP pathway in *Synechocystis*. The native enzymes relevant to the pathway are marked in red (along with the relevant genes and their IDs (in italics). The diagram is based on the pathway in the KEGG database (http://www.kegg.jp/kegg/, accessed on 22 September 2025). Abbreviations used: G3P, glyceraldehyde 3-phosphate; DXP, 1-deoxy-d-xylulose 5-phosphate; MEP, 2-C-methyl-d-erythritol 4-phosphate; DMAPP, dimethylallyl pyrophosphate; IPP, isopentenyl PP; GPP, geranyl PP; FPP, farnesyl PP; GGPP, geranylgeranyl PP; DXS, DXP synthase; DXR, DXP reductoisomerase; IspD, MEP cytidylyltransferase; IspE, ME-CDP kinase; IspF, ME 2,4-cyclodiphosphate synthase; IspG, (E)-4-hydroxy-3-methylbut-2-en-1-yl PP synthase; IspH, HMBPP reductase; IDI/*Ipi*, isopentenyl diphosphate isomerase; GPPS, GPP synthase; FPPS, FPP synthase; GGPPS, GGPP synthase.

Terpenoids represent one of the largest classes of natural products, widely utilized as pigments, antioxidants, flavor compounds, pharmaceuticals, and nutraceutical ingredients [1]. Industrial production of terpenoids traditionally relies on plant extraction or chemical synthesis, both of which face limitations, including land-use constraints, seasonal variability, low extraction yields, and environmental concerns [2]. Consequently, the development of sustainable bio-based production platforms has become increasingly important for securing a stable supply of high-value isoprenoids. In photosynthetic organisms, the 2-C-methyl-d-erythritol 4-phosphate (MEP) pathway, also called the non-mevalonate or non-MVA pathway, is the plastid/bacterial route for isoprenoid (terpenoid) precursor biosynthesis, namely isopentenyl pyrophosphate (IPP) and dimethylallyl pyrophosphate (DMAPP). It is ubiquitously present in many bacteria, cyanobacteria, and in the plastids of algae and plants [3]. In cyanobacteria and photoautotrophic algae, the MEP pathway is especially relevant because terpenoid and carotenoid metabolites, often derived from downstream prenyl-diphosphate intermediates such as geranyl PP (GPP), farnesyl PP (FPP), and geranylgeranyl PP (GGPP), are important both for photosynthesis (pigments, chlorophyll side-chains) and as industrially exploitable molecules [4]. Briefly, the pathway begins with the condensation of pyruvate + glyceraldehyde-3-phosphate (G3P) to form 1-deoxy-D-xylulose 5-phosphate (DXP) catalyzed by DXS. After several steps, it yields IPP and DMAPP (Figure 1). Those then serve as building blocks for prenyltransferases to build longer prenyl diphosphates, e.g., GGPP (C_20_) is formed from IPP + DMAPP via a GGPP synthase (GGPPS) [5]. In cyanobacteria, the MEP pathway has been engineered to produce isoprene, limonene, diterpenes, etc., in which the endogenous flux through the MEP pathway is a limiting factor [6]. Among various studies on cyanobacteria, notably in *Synechococcus elongatus* PCC 7942 and *Synechocystis* sp. PCC 6803 (hereafter *Synechocystis*), the MEP pathway was used to drive isoprene production from CO_2_ or by rerouting metabolism to generate non-native diterpenes (e.g., taxol precursors) by coupling the MEP pathway to GGPP synthesis [6,7]. GGPP serves as a branching precursor to a wide array of terpenoid classes, notably diterpenoids, carotenoids, chlorophyll side chains, tocopherols, and other isoprenoid derivatives, and manipulating GGPP flux may enable enhanced production of pigments in cyanobacteria or algae for nutrition, feed, cosmetics, and antioxidants [8]. Thus, boosting flux through the MEP pathway (and downstream GGPP) is of considerable biotechnological interest in algae and cyanobacteria. GGPP serves as a universal precursor for a wide range of high-value terpenoids with significant industrial applications. These include carotenoids, tocopherols (vitamin E), quinones, and diterpenoids, many of which are widely used in the pharmaceutical, nutraceutical, and cosmetic industries [9]. Notably, GGPP acts as a direct precursor for bioactive compounds such as paclitaxel (Taxol), an important anticancer drug, highlighting its relevance in pharmaceutical biotechnology. In addition, GGPP-derived terpenoids contribute to the production of natural pigments, fragrances, and flavoring agents, making it an essential metabolic intermediate for the fragrance and food industries [10]. Furthermore, GGPP is involved in protein prenylation, a critical modification linked to cellular signaling pathways and disease mechanisms, thereby underscoring its importance as a target for therapeutic development [11].

**Figure 2:**
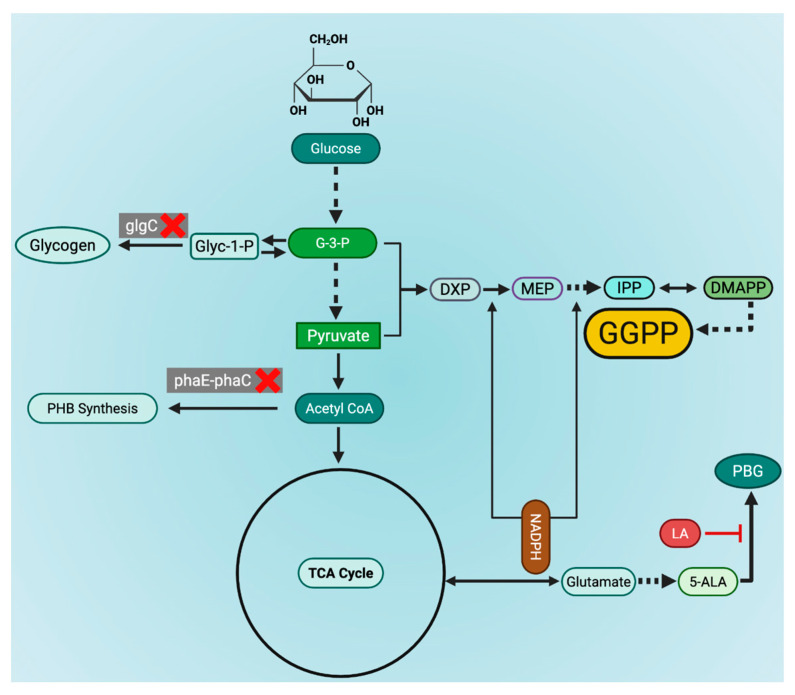
Comprehensive MEP pathway representing the linkage to glycogen, PHB and ALA synthesis in *Synechocystis*. The inactivated pathway genes are indicated with a red cross, and broken arrows represent the cascade reactions. The diagram is based on the pathway in the KEGG database (http://www.kegg.jp/kegg/, accessed on 22 September 2025). Abbreviations used (that are not explained in Figure 1): TCA, tricarboxylic acid; PHB, poly(3-hydroxybutyrate); NADPH, nicotinamide adenine dinucleotide phosphate; 5-ALA, 5-aminolevulinic acid; LA, levulinic acid; PBG, porphobilinogen.

In the model cyanobacterium *Synechocystis*, the majority of assimilated carbon is funneled through glycolysis and subsequently metabolized into major carbon reserve compounds, including glycogen (GL), poly(3-hydroxybutyrate) (PHB), lipids, and intermediates of the tricarboxylic acid (TCA) cycle that further serve as precursors for various biosynthetic routes, such as the 5-aminolevulinic acid (ALA) and γ-aminobutyric acid (GABA) shunt pathways [12,13]. Recent studies have attempted to elucidate the metabolic interconnections among GL, PHB, GABA, and ALA in *Synechocystis*. These studies have partially demonstrated that disrupting one of these biosynthetic pathways can redirect carbon flux toward alternative carbon-reserve synthesis pathways. In this context, disruption of the GL and PHB pathways has been investigated to assess its impact on GABA and ALA biosynthesis previously [12]. To further understand how GL and PHB disruption influences the MEP pathway, the *Synechocystis* mutants were constructed by inactivating the ADP-glucose pyrophosphorylase (ADP-GPP) (*glg*C; gene ID: *slr1176*) and polyhydroxyalkanoate synthase (PHAS) (*pha*E-*pha*C operon; gene IDs: *slr1829*-*slr1830*) genes, which correspond to the catalysis of GL and PHB [14]. To inactivate GL synthesis, the plasmid pGLGC::CmR harboring a 1651-bp fragment, comprising the interrupted *glg*C coding region with chloramphenicol-resistant gene from pUC57, was transformed into *Synechocystis*. Conversely, the interruption of PHAS was done by deleting the part of coding region of the *pha*E-*pha*C operon and replacing by the spectinomycin resistant gene of pHP45Ω to generate the plasmid pPHA-EC::SpcR to transform into *Synechocystis* [14]. Mutants were screened by PCR-based genotyping and further confirmed by sequencing to ensure complete segregation. Previously, mutant strains ΔP (deficient in PHB synthesis) and ΔGP (deficient in both GL and PHB synthesis) have been analyzed to assess their effects on GABA and ALA metabolism [12,13]. In the present study, the disruption of GL and PHB synthesis in *Synechocystis* was examined in relation to MEP pathway regulation and GGPP synthesis (Figure 2). Furthermore, the effects of various physicochemical factors on photosynthetic pigment accumulation, GGPP levels, and gene expression were analyzed to uncover the molecular mechanisms underlying MEP pathway regulation in connection with neighboring carbon metabolism, an area that remains poorly understood. While previous studies have explored metabolic engineering strategies targeting the isoprenoid biosynthetic pathway, including gene knockouts and pathway optimization to enhance terpenoid production, most efforts have focused on single-gene modifications or heterologous pathway reconstruction in model organisms such as *Escherichia coli* and yeast [15,16]. In cyanobacteria, engineering of the MEP pathway has been attempted, but improvements in isoprenoid precursors such as GGPP remain limited due to tight metabolic regulation and competing carbon fluxes [6,17]. In the present study, targeted disruption of carbon-storage pathways (ΔP and ΔGP) was combined with selected physiological interventions to investigate their coordinated effects on carbon partitioning toward GGPP biosynthesis. While similar combinations of genetic and cultivation-based approaches have previously been applied to enhance other terpenoids in cyanobacteria, their impact on GGPP supply has not been specifically examined. Therefore, this work advances current knowledge by providing new insights into coordinated carbon partitioning toward GGPP and establishes an effective framework for improving isoprenoid precursor production in photosynthetic systems that has not been clearly demonstrated in earlier reports. Davies et al. demonstrated improved production of a heterologous terpenoid in a single storage-pathway mutant of *Synechocystis*, highlighting the importance of carbon partitioning for metabolic engineering. Building upon this concept, the present study systematically investigates a double knockout strain (ΔGP) to evaluate enhancement of the endogenous GGPP pool and its associated native pigments under a range of physiologically relevant growth conditions [17]. This multi-condition analysis provides new evidence that simultaneous disruption of carbon-storage pathways can serve as a general metabolic engineering strategy for increasing intrinsic terpenoid precursor availability in *Synechocystis*. Metabolic modelling studies in cyanobacteria have demonstrated that reactions leading to GGPP formation are strongly correlated with downstream terpenoid productivity [4,5,18]. Consequently, engineering cyanobacterial strains with enhanced GGPP flux could provide a robust chassis for modular terpenoid biosynthesis, offering promising applications in the development of bio-based routes for advanced biofuels and the sustainable production of high-value pigments and antioxidants for nutraceutical and cosmetic industries.

## 2. Results and Discussion

GGPP serves as a central metabolic intermediate that integrates carbon flux from primary metabolism toward the formation of light-harvesting and photoprotective pigments essential for photosynthesis and oxidative stress defense [19,20]. Rational metabolic engineering strategies (Table 1), such as introducing mutations in enzymatic pathways or disrupting carbon-storage pathways, have been shown to enhance precursor availability and improve terpenoid production in cyanobacteria by redirecting reducing power and carbon skeletons toward the MEP pathway [7,21,22].

The present study investigated the effects of carbon redistribution pigment accumulation and MEP pathway regulation in *Synechocystis* wild-type (WT) and the engineered mutants ΔP and ΔGP. The strains were subjected to glucose supplementation, dodecane treatment, levulinic acid exposure, and UV irradiation to evaluate the combined influence of genetic modifications and environmental factors on pigment production, gene expression, and GGPP biosynthesis. UVR was of particular interest, as it is known to induce photoprotective pigment synthesis and activate MEP pathway gene expression in *Synechocystis* [26]. The physiological parameters were selected based on prior literature and preliminary optimization experiments. In previous studies, these parameters were optimized to balance cellular growth, metabolic activity, and product recovery and were shown to support enhanced terpenoid and pigment production without inducing significant cytotoxic effects in *Synechocystis* [12,13,14]. Therefore, in the present study, we maintained these experimentally validated conditions to ensure comparability and reproducibility while specifically focusing on improving GGPP supply. This experimental framework provides an integrated view of how environmental cues and genetic modifications synergize to regulate GGPP biosynthesis and isoprenoid metabolism in cyanobacteria.

### 2.1. Contrasting Impacts of Various Physicochemical Factors on Biomass and Pigment Levels in Synechocystis Strains

**Figure 3:**
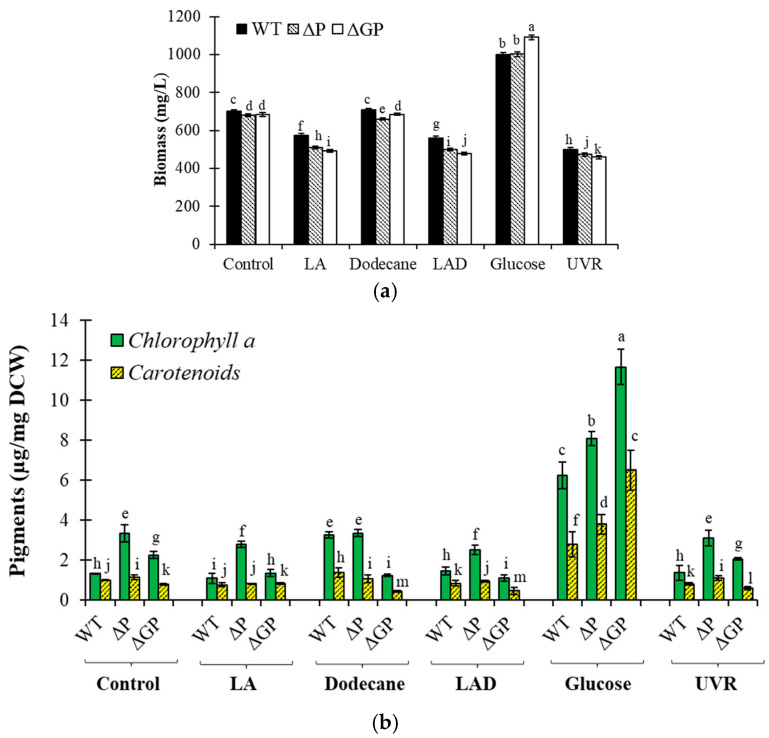
Growth and pigment analysis: (**a**) Growth of cultures (biomass levels of *Synechocystis* WT and mutant strains). (**b**) Pigments (chlorophyll *a* and total carotenoid concentration) of *Synechocystis* WT and mutant strains at the late log phase of growth under control (untreated), levulinic acid (LA), dodecane, LA + dodecane (LAD), glucose, and UVR treatment. Different letters on each column indicate the significant differences at a 95% significance level, according to Duncan’s multiple range test (*p* ≤ 0.05), while identical letters denote no significant difference.

Under control/untreated conditions (BG11 medium without any additional supplements or treatments), all strains exhibited comparable biomass, indicating that the mutations do not significantly affect basal growth (Figure 3a). However, under stress or inhibitory conditions such as LA, LAD, and UVR, both mutants showed a greater reduction in biomass than WT, suggesting that ΔP and especially ΔGP are more sensitive to environmental stress. In the presence of dodecane, biomass remained relatively similar across strains, although a slight reduction in ΔP and ΔGP indicates mild sensitivity to solvent stress. In contrast, glucose supplementation significantly enhanced biomass in all strains, with ΔGP showing the highest increase, suggesting improved carbon utilization or metabolic efficiency in this mutant.

Pigment quantification revealed distinct differences among *Synechocystis* strains under varying physiological conditions (Figure 3b). Both mutants (ΔP and ΔGP) exhibited significantly higher chlorophyll *a* and carotenoid accumulation than the WT, particularly under glucose supplementation. The ΔGP strain demonstrated the greatest enhancement, reaching approximately 12 µg mg^−1^ dry cell weight (DCW) chlorophyll *a* and 6 µg mg^−1^ DCW carotenoids, suggesting improved carbon flux toward the isoprenoid pathway. This agrees with reports indicating that increased carbon availability enhances flux through the MEP pathway, favoring pigment biosynthesis [6,15]. Although growth was also monitored through OD_730_ measurements over the cultivation period (Supplementary Figure S1), the pigment accumulation in this study represents an endpoint phenotype and does not directly provide information on growth kinetics or cellular fitness under the applied conditions. Hence, it should be interpreted in the context of overall physiological responses of the engineered strains. Exposure to LA caused a decrease in chlorophyll *a* content in all strains compared with the control, whilst carotenoid pigment level remained unaffected. LA’s role is established as an inhibitor of ALA dehydratase, which blocks the conversion of ALA to porphobilinogen (Figure 2), a key step in the tetrapyrrole pathway leading to chlorophyll biosynthesis [12,27]. Our findings are consistent with earlier studies showing that LA supplementation inhibits microalgal growth, likely due to its suppressive effect on chlorophyll biosynthesis [13,28]. This is supported by the concomitant decrease in the growth of strains as discussed earlier (Figure 3a). It also indicates the upregulation of the MEP pathway for compensatory pigment (carotenoids) synthesis due to ALA pathway inhibition under LA treatment. Notably, the ΔP mutant consistently outperformed ΔGP in response to LA and dodecane treatments, implying that the redirection of carbon from PHB synthesis more effectively supports terpenoid precursor production than blocking the glycogen formation. However, in contrast to the control, dodecane treatment led to both increased and decreased pigment levels in WT and mutants, respectively, without affecting the viability or growth of the strains (Figure 3a). Dodecane is known to act as a biocompatible organic solvent that facilitates “milking” or in situ extraction of hydrophobic metabolites in phototrophs. This process can induce mild stress responses, stimulating pigment biosynthesis to maintain photosynthetic capacity, as observed in our study on the *Synechocystis* WT strain [29,30]. The strong pigment induction observed here suggests that dodecane either triggers adaptive upregulation of photosynthetic pigments or facilitates pigment stabilization and extraction. However, despite the possible redirection of carbon flow toward the MEP pathway in ΔGP, low pigment levels in dodecane-treated cells indicate impaired regulatory mechanisms of pigment biosynthesis, warranting further investigation.

There was no significant difference found between pigment levels in *Synechocystis* strains as compared to the control in response to the less detrimental UV-A, suggesting that the genetic redirection synergizes with UV-induced signaling to maximize protective pigment production. Previous studies have reported contrasting effects of different UV wavelengths on cyanobacterial pigments; for example, UV-B exposure has been associated with reductions in photosynthetic pigment levels in *Synechocystis* [31], whereas UV-A exposure has been reported to stimulate carotenoid accumulation in some cyanobacterial systems due to their photoprotective antioxidant functions. Carotenoids can quench singlet oxygen and dissipate excess excitation energy, thereby preventing photodamage to photosystem reaction centers in cyanobacteria [19,32]. We further investigated the effects of physiological conditions on the gene expression levels in the MEP pathway to gain deeper insight into the genetic mechanisms governing metabolite fluctuations in these strains.

### 2.2. Expression Profiling of MEP Pathway Genes in Synechocystis WT and Mutant Strains

**Figure 4:**
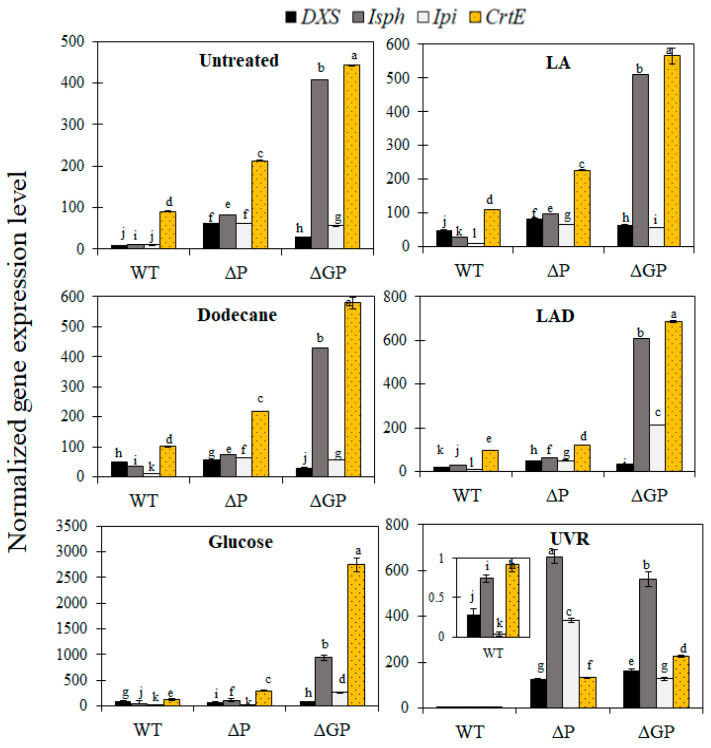
Transcriptional regulation of MEP pathway genes in *Synechocystis* strains under various treatments, using BG11 medium as a control (untreated). Results were normalized to *16S* rRNA transcript levels, and bars represent the mean ± standard deviation of three independent biological replicates, each analyzed in technical triplicate. If the error bar cannot be seen, the deviation is very small. Different letters on each column indicate the significant differences at a 95% significance level, according to Duncan’s multiple range test (*p* ≤ 0.05).

The transcriptional analysis of the MEP pathway genes reveals significant upregulation in both mutant strains (ΔP and ΔGP) compared to the WT (Figure 4). Among the genes analyzed, *Isph* and *CrtE* transcripts show the highest abundance, particularly in the ΔGP mutant, where expression levels exceed 400% relative to WT. These genes encode 4-hydroxy-3-methylbut-2-enyl diphosphate reductase and GGPP synthase, which catalyze key downstream steps leading to GGPP formation and the synthesis of isoprenoid precursors for carotenoids and chlorophylls [4]. Their elevated expression suggests possibly enhanced flux through the MEP pathway in ΔGP, likely supporting increased pigment biosynthetic capacity under certain conditions. Moderate elevation of *DXS* and *Ipi* transcripts, which are considered major flux-controlling nodes [33], in ΔP and ΔGP also indicates a coordinated upregulation of the pathway, potentially as a compensatory response to metabolic or regulatory disruptions caused by the gene deletions. In contrast, the WT maintains relatively low transcript levels across all MEP genes, consistent with basal isoprenoid metabolism under standard growth conditions. In plants, *DXS* transcripts are regulated by feedback mechanisms involving IPP/DMAPP and by external environmental signals. On the other hand, the overexpression of DXR in *Arabidopsis* increases flux through the MEP pathway but does not feedback to upregulate *DXS* transcription, suggesting separable regulatory layers. Beyond transcription, DXS is also regulated post-transcriptionally and post-translationally, suggesting that the regulation of MEP-pathway genes is complex, involving transcriptional, post-transcriptional, and feedback mechanisms [33,34]. Overall, these data imply that mutations in ΔP and ΔGP may relieve feedback inhibition or activate transcriptional regulators that enhance isoprenoid precursor biosynthesis, providing a mechanistic basis for the altered pigment phenotypes observed.

Under LA and dodecane, all strains showed upregulation of MEP pathway transcripts compared to untreated controls, with ΔGP exhibiting the highest transcript accumulation (Figure 4). The upregulation was most evident in *Isph* and *CrtE*, suggesting that downstream isoprenoid flux was particularly affected. However, this transcriptional response was not accompanied by a corresponding increase in pigment accumulation under LA treatment. This transcript–metabolite disconnect may reflect regulation beyond gene expression, such as altered enzyme activity, post-translational control, or feedback inhibition by isoprenoid intermediates (IPP/DMAPP), as reported for terpenoid pathways in previous studies [33,34], and requires further confirmation by determining relevant enzyme activities. The strong induction of *CrtE*, which encodes GGPP synthase, suggests enhanced commitment toward GGPP biosynthesis under solvent exposure. This response was attenuated in WT but amplified in ΔP and ΔGP, indicating altered regulatory sensitivity in these mutants. When LA and dodecane were combined (LAD), overall transcript levels were intermediate between the individual treatments. This suggests that dodecane partially alleviates the inhibitory effect of LA, possibly by stimulating stress-responsive transcription or stabilizing pigments through extraction, though LA’s block on ALA conversion still restricts maximal expression.

The glucose-supplemented condition led to dramatic upregulation of MEP genes, especially *CrtE* (>22-fold increase in ΔGP), suggesting that mixotrophic conditions strongly stimulate isoprenoid biosynthesis-enhancing carbon flux and reducing power (NADPH). Exogenously supplied glucose enters central metabolism through glycolysis and likely enhances carbon flux into the MEP pathway (as shown in Figure 2) by increasing the supply of G3P and pyruvate, the precursors of DXS activity [35]. The exceptionally high transcript levels in ΔGP suggest that this mutant has overactive regulation of carbon partitioning toward terpenoid synthesis. This was further validated by quantifying intracellular GGPP levels as described in the next section. Under UVR, MEP pathway genes were also upregulated (Figure 4), particularly *Isph* and *CrtE*, with higher induction in mutants than in WT. UV exposure is known to stimulate carotenoid synthesis as a photoprotective response, enhancing antioxidant pigment production to counteract photooxidative stress [36]. The increased *Isph* expression under UVR thus reflects activation of protective isoprenoid metabolism, more pronounced in mutants, possibly due to their altered stress regulation.

### 2.3. Effect of Physiological Conditions on MEP Pathway Regulation and GGPP Accumulation in Synechocystis Mutants

**Figure 5:**
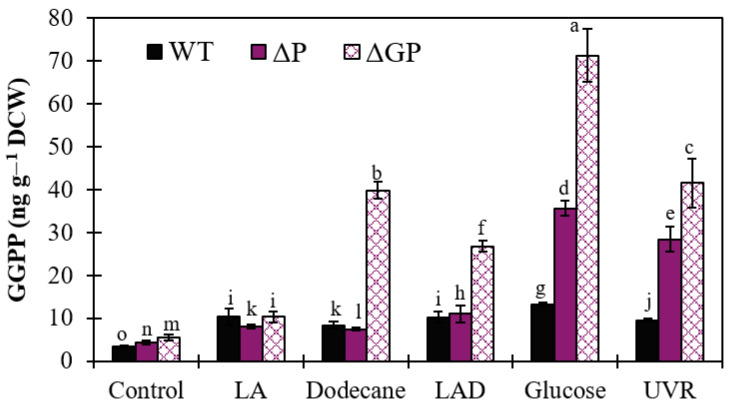
Intracellular levels of GGPP in *Synechocystis* WT, ΔP, and ΔGP strains under various treatments. BG11 medium was used as a control (untreated). Results represent the mean ± standard deviation (SD) of experiments conducted in three biological replicates (*n* = 3), and the error bars denote the SD values. Different letters on each column indicate the significant differences at a 95% significance level, according to Duncan’s multiple range test (*p* ≤ 0.05).

**Figure 6:**
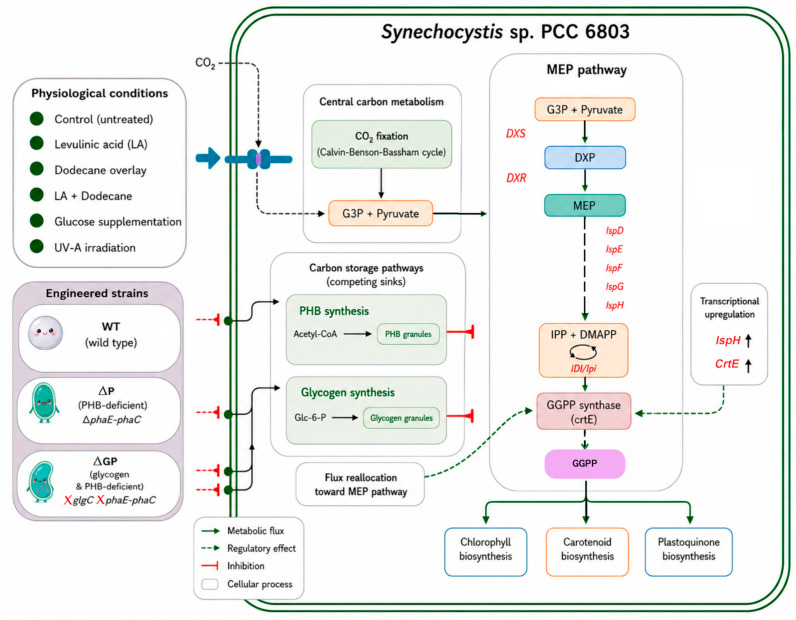
Proposed model summarizing metabolic rewiring and physiological regulation of GGPP biosynthesis in engineered *Synechocystis* sp. PCC 6803. Deletion (indicated by red crosses) of glycogen (*glgC*) and PHB (*phaE–phaC*) biosynthetic pathways redirects carbon toward the MEP pathway, resulting in increased expression of *Isph* and *CrtE* and enhanced GGPP accumulation under selected physicochemical treatments. Hashed black arrows indicate cascade reactions.

In agreement with transcript and pigment data, intracellular GGPP levels peaked in the ΔGP strain under glucose and UVR treatments, reaching ~71 and 42 ng g^−1^ DCW, respectively (Figure 5). LA alone has little effect, but when combined with dodecane (LAD), it moderately enhances GGPP levels. These findings are consistent with the interpretation that metabolic rewiring through disruption of glycolysis and PHB biosynthesis may enhance carbon partitioning toward isoprenoid biosynthesis. Moreover, the positive effect of glucose suggests that enhanced reductant and ATP supply reinforce MEP pathway flux, as previously demonstrated in photoheterotrophic cyanobacteria [21]. The results showed that UVR not only triggers defensive pigment biosynthesis but also enhances flux through the MEP-derived GGPP pool with a concomitant increase in pathway gene expression, which serves as a shared precursor for carotenoids and chlorophylls. The improved pigment accumulation in ΔGP indicates that blocking glycogen and PHB synthesis increases carbon and NADPH availability for the MEP pathway, thereby reinforcing the organism’s intrinsic photoprotective response [6,31]. Interestingly, dodecane supplementation led to increased intracellular GGPP levels in ΔGP (>7-fold higher than control). This might be attributed to two main effects: (1) enabling effective removal or sequestration of hydrophobic downstream metabolites (reducing feedback inhibition and toxicity) and/or (2) preserving downstream demand for GGPP conversion (thus pulling flux through the MEP pathway), as demonstrated previously in few algal species where dodecane overlay extraction method helped to avoid possible product-induced feedback inhibition [17,37]. The enhanced flux capacity of the mutant then manifests as higher intracellular GGPP levels under dodecane treatment. Similar metabolic redirection strategies have been shown to enhance terpenoid yields in cyanobacteria and *E. coli* by overexpressing MEP pathway genes or limiting competing carbon sinks [25,26]. Nonetheless, under dodecane treatment, the increased GGPP in ΔGP, concurrent with reduced chlorophyll and carotenoid content (Section 3.1), suggests a pronounced metabolic decoupling between precursor supply and downstream pigment biosynthesis. The observed pattern may indicate a metabolic bottleneck or feedback regulation, resulting in GGPP accumulation without efficient conversion to downstream products under dodecane treatment. It may also arise from a combination of redox and energy imbalances that limit biosynthetic capacity, together with stress-induced inhibition of pigment biosynthetic pathways that is needed to confirm by targeted metabolomic analyses of carotenoid pathway intermediates. Though dodecane is biocompatible with no apparent stress induced on *Synechocystis* (as earlier discussed in Section 3.1), the importance of coordinated regulation between precursor supply, cellular energy balance, and downstream pathway capacity in ΔP and ΔGP mutants requires further investigation. In addition to GGPP accumulation, the removal of glycogen and PHB synthesis in the ΔGP likely redirects excess fixed carbon toward multiple alternative metabolic sinks, which collectively influence the observed decrease in pigment levels and overall carbon balance. Along with intracellular redistribution, excess carbon may also be dissipated through overflow metabolism and metabolite excretion. When glycogen synthesis is impaired, cyanobacteria have been shown to excrete central metabolites such as pyruvate, 2-oxoglutarate, succinate, and malate as a mechanism to prevent intracellular carbon overload [38]. This excretion represents a loss of fixed carbon that would otherwise be available for biosynthetic pathways, thereby contributing to reduced pigment accumulation despite increased precursor availability. Additionally, as demonstrated in a previous study [12], disruption of these storage pathways also leads to a significant redistribution of carbon flux toward the TCA cycle and associated metabolites including GABA and ALA. Organic acid profiling in these mutants confirmed increased flux into TCA intermediates, accompanied by upregulation of genes involved in central carbon metabolism. Overall, this study demonstrates for the first time that targeted deletions of carbon-storage pathways can effectively enhance GGPP and pigment production in cyanobacteria. The observed increase in GGPP represents a relative enhancement of a naturally low-flux pathway, rather than complete redirection of carbon from storage pathways. The ΔP and ΔGP mutants exhibited enhanced transcriptional responses, suggesting that the genetic modifications alter the regulatory control of isoprenoid metabolism. These changes may increase the potential for pigment production under specific stress or nutrient conditions. A schematic overview of the proposed metabolic and regulatory interactions underlying these responses is presented in Figure 6. Collectively, these findings suggest that the engineered strains represent a promising chassis for the future development of photosynthetic production platforms for carotenoids, chlorophyll derivatives, and other terpenoid-based compounds, although further optimization and scale-up studies are required to assess their industrial feasibility.

## 3. Materials and Methods

### 3.1. Strains and Growth Conditions

The WT strain of *Synechocystis* was obtained from the Pasteur Culture Collection and cultivated in Blue-Green (BG11) medium supplemented with 20 mM [4-(2-hydroxyethyl)-1-piperazineethanesulfonic acid]-NaOH (pH 7.5) under standard photoautotrophic conditions [39]. Its derivative mutants, ΔP (knockout of the *pha*E–p*ha*C operon) and ΔGP (knockout of *glgC* and the *pha*E–*pha*C operon), were previously constructed and maintained in BG11 medium supplemented with the appropriate antibiotics [14]. The ΔP mutant was maintained with spectinomycin (20 μg mL^−1^), while the ΔGP mutant was maintained with chloramphenicol (30 μg mL^−1^) and spectinomycin (20 μg mL^−1^) (Research Products International Corp S2300025.0). All strains were grown at 28 ± 2 °C with added Na_2_CO_3_ as a carbon source (0.2 mM), under continuous white light at 50 μmol photons m^−2^ s^−1^, with constant shaking at 160 rpm. The growth rate was determined by measuring the optical density (OD) at 730 nm (OD_730_) (Supplementary Figure S1). For growth on solid medium, cultures were maintained on BG11 agar plates (1.5% agar, Difco^TM^) supplemented with 10 mM N-[tris(hydroxymethyl)methyl]-2-aminoethanesulfonic acid (pH 7.5) and 3 g L^−1^ sodium thiosulfate, with or without the corresponding antibiotics for mutant or WT strains, respectively.

### 3.2. Experimental Treatments

Cyanobacterial cultures were subjected to various physicochemical treatments as alternative growth conditions to investigate metabolite biosynthesis associated with the deleted pathways in the mutant strains. *Synechocystis* WT and mutant cultures were first grown in 200 mL of BG11 medium to the late logarithmic phase (under basic culture conditions as stated in 2.1), after which levulinic acid (LA) and glucose were added to final concentrations of 40 and 10 mM, respectively (as established earlier [12,13]). An amount of 5 mL of filter-sterilized n-dodecane overlay was applied to the cultures, whereas LA-supplemented cultures with additional dodecane (LAD) were also studied. Under all treatment conditions, cultures were incubated for an additional 72 h. For UV exposure, late-log-phase WT and mutant cultures were irradiated with UV-A (UVR) (315–400 nm) with 10 W m^−2^ intensity for 2 h prior to subsequent analyses.

### 3.3. Extraction of Metabolites and Analyses

Cyanobacterial cultures grown under the different physicochemical conditions were collected by centrifugation at 2500× *g* for 10 min at room temperature, followed by two washes with ice-cold double-distilled water prior to metabolite extraction. Pigment analysis (chlorophyll *a* and total carotenoids) of *Synechocystis* WT and mutant strains was performed using an *N*, *N*-dimethylformamide (DMF) extraction method adapted from [13]. In brief, liquid cultures of both WT and mutant strains were initiated at an OD_730_ of approximately 0.1 and grown to the late logarithmic phase (OD_730_ of 0.8–1.0) before exposure to the experimental treatments described in Section 2.2. Cell suspensions equivalent to 10 mg of biomass from the different growth phases were centrifuged at 2500× *g* for 10 min, and the resulting pellets were extracted with DMF under vigorous mixing at room temperature. The chlorophyll *a* and carotenoid contents were then quantified from the DMF extracts.

GGPP extraction from *Synechocystis* WT and mutant cells was performed by vigorously vortexing the biomass with 500 μL of 80% methanol. The mixture was incubated for 2 h, followed by centrifugation at 9800× *g* for 10 min at 4 °C. The resulting supernatant was collected and evaporated to dryness using a vacuum concentrator (Labconco Corporation, Kansas City, MO, USA) at 30 °C. The dried residue was subsequently resuspended in a water:chloroform mixture (5:1, *v*/*v*), mixed thoroughly, and centrifuged at 6200× *g* for 10 min. The aqueous phase was recovered, combined with methanol, and filtered through a 0.45 μm Millipore membrane prior to analysis. Quantification of GGPP was conducted using liquid chromatography-triple quadrupole mass spectrometry (LC-QQQ-MS; TSQ Altis™ Plus, Thermo Fisher Scientific, Waltham, MA, USA). Chromatographic separation was achieved on a Shim-pack Scepter C18-120 column (3 µm, 2.1 × 100 mm) (Shimadzu Corporation, Kyoto, Japan) using 5 mM NH_4_HCO_3_ as solvent A and acetonitrile as solvent B. The gradient program started at 0% B (100% A), increased linearly to 100% B over 8 min, held at 100% B for 2 min, and then returned to 0% B for 2.5 min before the next injection. The flow rate was maintained at 0.4 mL min^−1^, and the column temperature was set to 40 °C. Mass spectrometric detection was performed in Multiple Reaction Monitoring (MRM) mode with a positive-ion electrospray ionization (ESI) source. GGPP was identified based on retention time and characteristic ion transitions using an authentic GGPP standard (Sigma-Aldrich, Singapore). Quantification was performed using a single-point external calibration approach, where a known concentration of the GGPP standard obtained from Sigma-Aldrich, St. Louis, MO, USA, was used to estimate sample concentrations. Data acquisition and processing were carried out using Thermo Scientific™ Xcalibur™ software (version 4.8, Thermo Fisher Scientific, Waltham, MA, USA) and Thermo TraceFinder software (version 5.1, Thermo Fisher Scientific, Waltham, MA, USA).

### 3.4. Gene Expression Analysis by RT-qPCR

For gene expression analysis, *Synechocystis* WT and mutant cultures were grown under the experimental conditions outlined in Section 2.2 and collected by centrifugation at 2500× *g* for 10 min. Total RNA was extracted from the resulting cell pellets, and reverse transcription–quantitative PCR (RT-qPCR) was performed according to the protocols described by Kanwal and Incharoensakdi [14]. The cDNAs obtained from the WT, ΔP, and ΔGP strains were used to quantify target gene transcript levels by employing real-time PCR assays. Corresponding primer sequences and their respective target gene IDs are listed in Table 2. The optimized real-time PCR reaction mixture contained 1 μL of cDNA, a final concentration of 0.4 μM for each primer, 5 μL of 2× QPCR Green Master Mix (LRox, biotechrabbit, Singapore), and nuclease-free water, adjusted to a final volume of 10 μL. Real-time PCR was carried out using a CFX96 Touch™ Real-Time PCR Detection System (Bio-Rad, Singapore) with the following thermal profile: an initial activation step at 95 °C for 2 min, followed by 40 amplification cycles of 95 °C for 5 s for denaturation and 60 °C for 30 s for annealing and extension. The 16S rRNA gene was used as the internal reference, and DNase-treated RNA samples without reverse transcriptase were included as non-template controls to confirm the absence of genomic DNA contamination. Data acquisition was performed using Bio-Rad CFX Manager™ software (version 3.0), and relative gene expression levels were calculated using the 2^−ΔΔCT^ method [12]. All RT-qPCR analyses were performed using three independent biological replicates, each analyzed in technical triplicate.

### 3.5. Statistics

All results are presented as the means of three independent experimental replicates, with error bars representing one standard deviation (SD). Statistical analyses were carried out using one-way analysis of variance (ANOVA), and mean comparisons were performed by using Duncan’s multiple range test at a significance threshold of *p* ≤ 0.05 (95% confidence level). Different superscript letters above the bars in each figure indicate statistically significant differences, while identical letters denote no significant difference. The highest mean in each dataset is marked with the letter “a.” All statistical analyses were performed using SPSS (version 15.0).

## 4. Conclusions

The present study elucidates the interconnected regulation of the MEP pathway, pigment biosynthesis, and GGPP accumulation in *Synechocystis* through combined genetic and physiological modulation. The targeted deletions of PHB and glycogen biosynthetic pathways (ΔP and ΔGP) effectively redirected carbon flux toward isoprenoid metabolism, enhancing both pigment and GGPP levels. Among the tested conditions, glucose supplementation and UV-A irradiation most strongly stimulated the MEP pathway, underscoring the role of carbon availability and photooxidative stress in activating terpenoid precursor synthesis. The ΔGP mutant exhibited the most pronounced metabolic response, displaying elevated *Isph* and *CrtE* expression and a corresponding increase in GGPP content, findings that are consistent with enhanced flux through the MEP pathway. The effect of dodecane further highlighted that the solvent overlay likely alleviated product feedback inhibition and facilitated in situ extraction of hydrophobic terpenoids, resulting in a marked elevation of intracellular GGPP, particularly in ΔGP. These findings reinforce the notion that metabolic rewiring, combined with controlled physicochemical stimuli, can synergistically enhance precursor and pigment production in cyanobacteria. From a biotechnological perspective, these results position the ΔGP strain as a promising chassis for sustainable biosynthesis of high-value isoprenoids. For enhanced GGPP production, one must couple carbon-supply rerouting with enhancing MEP pathway capacity (e.g., *DXS*, *Ipi* overexpression), improving GGPP synthase, ensuring downstream sinks, and maintaining robust host physiology. Future studies should focus on (i) metabolic flux analysis and isotope labeling to quantify carbon redistribution within the MEP pathway, as advanced metabolomic approaches, including IPP/DMAPP quantification and ^13^C-based metabolic flux analysis, will be essential to precisely resolve carbon partitioning within the MEP pathway; (ii) integrating promoter engineering or CRISPR-based fine-tuning of key genes such as *CrtE* and *Isph*; and (iii) exploring solvent-assisted bioprocess strategies for continuous extraction of terpenoids. Moreover, combining genetic optimization with environmental triggers such as light quality or carbon supplementation could further enhance yields of chlorophyll derivatives, carotenoids, and other GGPP-derived metabolites. Conversely, additional investigation on detailed photo-physiological parameters and elemental composition (C/N) could further elucidate physiological performance and carbon allocation dynamics. In addition, future studies should include complemented strains and marker-free mutants to unequivocally attribute the observed phenotypes to the targeted gene deletions and to exclude potential physiological effects arising from the antibiotic-resistance cassettes used during strain construction. Such integrated approaches may contribute to the future development of cyanobacteria as versatile platforms for more sustainable production of photosynthetic pigments and industrially relevant terpenoids, although further evaluation through metabolic flux analysis, scale-up studies, and techno-economic and sustainability assessments will be required.

## Figures and Tables

**Table 1:** Selected published engineering studies in cyanobacteria that relate (directly or indirectly) to enhancing precursor flux, such as GGPP or terpenoid production (which uses GGPP, FPP, or GPP) via the MEP pathway.

Host (Cyanobacterium)	Product/Target Compound	Key Engineering Strategy (Relevant to GGPP/MEP)	Titer or Outcome	Reference
*Synechocystis* sp. PCC 6803	Taxadiene (a diterpene, downstream GGPP)	Overexpression of heterologous GGPPS + taxadiene-synthase (TASY), integration into genome to divert native MEP → GGPP → diterpene.	~0.24 mg L^−1^ taxadiene after 7 days in shake-flask.	[7]
*Synechocystis* PCC 6803	13R-manoyl oxide (a diterpenoid)	Study of responses to heterologous production; attempted overexpression of upstream GGPP biosynthesis enzymes.	2 mg L^−1^ (~0.98 mg g^−1^ DCW) after 2 days of growth	[18]
*Synechococcus elongatus* UTEX 2973	Limonene (monoterpene)	Combinatorial metabolic engineering: including a beneficial mutation in the GGPP synthase gene (*CrtE*) to increase flux, upstream MEP pathway optimization.	~16.4 mg L^−1^ limonene; ~8.2 mg L^−1^ day^−1^.	[20]
*Synechocystis* PCC 6803	Valencene (sesquiterpene)	Deletion of competing pathways (sqs, shc), down-regulation of *CrtE* (GGPP synthase) to redirect FPP toward sesquiterpene, operon/fusion constructs with modulators.	~19 mg g^−1^ DCW after 48 h.	[23]
*Synechocystis* PCC 6803	Bisabolene (a terpenoid)	Sequential expression: overexpress native pathway bottlenecks (DXS, IDI) for increased precursor supply.	>180 mg L^−1^ culture after 10 days of cultivation.	[24]
*Synechocystis* sp. PCC 6803	Limonene (monoterpene)	Expression of a heterologous limonene synthase and *Abies grandis* GPPS, combined with overexpression of pentose phosphate pathway genes (*rpi* and *rpe*) to enhance precursor supply to the MEP pathway.	6.7 mg L^−1^ limonene (2.3-fold increase over the parental production strain).	[25]

Note: Reported titers and productivities were reproduced from the cited references and are expressed in the original units used by each study (e.g., mg L^−1^, mg g^−1^ DCW, and mg L^−1^ day^−1^). These values are provided for qualitative comparison only and should not be interpreted as directly quantitatively comparable across studies.

**Table 2:** Primers used in RT-qPCR. Gene annotations as per data available from CyanoBase [40]. (https://bio.tools/cyanobase 15 July 2025).

Gene Name/ID	Primer Sequences (5′ → 3′)	Product Size (bp)
*DXS*/*sll1945*	F-GGCGATGGTGCTTTAACC R-CTGCTGAGGCGGACTTTAT	161
*Isph*/*slr0348*	F-TTAATACCATTTGCGATGCC R-CCGCAATTTCTTGGAGATG	132
*Ipi*/*sll1556*	F-TAGCCTTCACCTATCAAGTCCG R-TCCTGGAGGGGATTGAGAT	163
*CrtE*/*slr0739*	F-GGACAAGTTCTAGACCTGGAATC R-GCCAGTCTCTGTTGTTGTTCC	158

Sequences of primers used in real-time PCR reactions for 16*S* were those described in a previous study [41].

## Data Availability

The original contributions presented in this study are included in the article/Supplementary Materials. Further inquiries can be directed to the corresponding author.

## Supplementary Materials

The following supporting information can be downloaded at: https://www.mdpi.com/article/10.3390/ijms27156680/s1.
