# Supplementary material for: Enhancing Geranylgeranyl Pyrophosphate Supply in Engineered Cyanobacteria for Improved Production of Natural Pigments and Terpenoid Precursors
Source: Int J Mol Sci. 2026 Jul 27;27(15):6680. doi: 10.3390/ijms27156680 (PMC13467309; doi:10.3390/ijms27156680)

Figure S1. Comparison of growth by measuring the optical density of cultures at 730 nm. Results represent the mean ± standard deviation (SD) of experiments conducted in triplicate (n = 3).

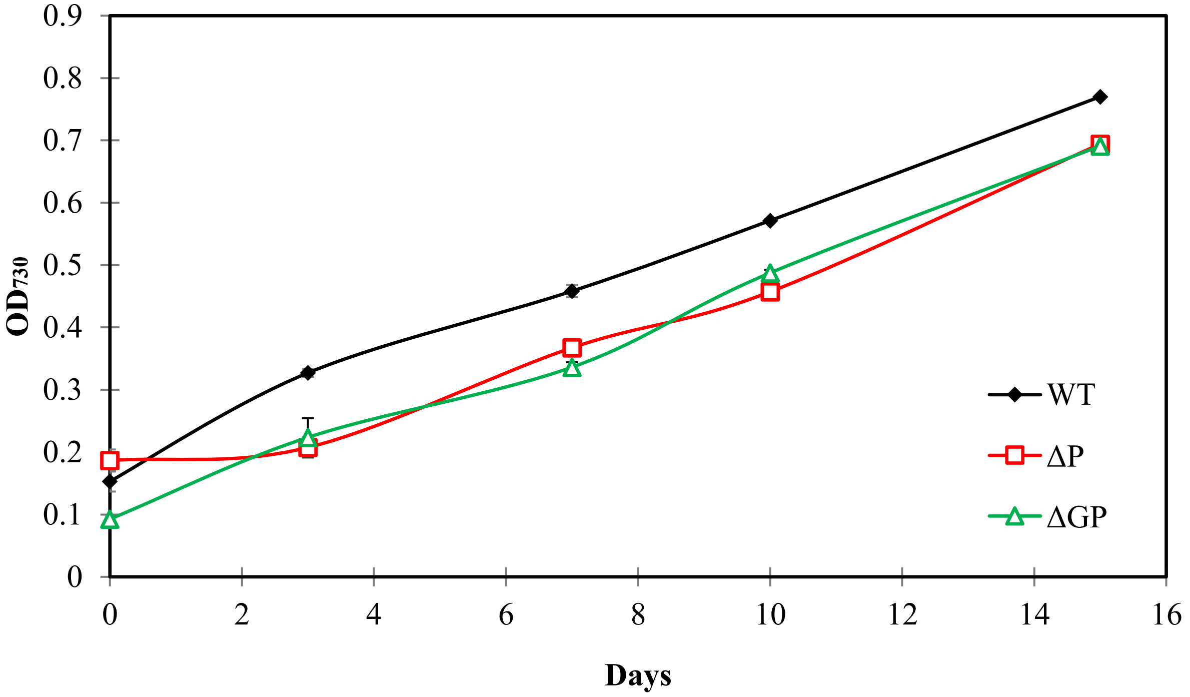

Supplement: Supplementary file 1 [file ijms-27-06680-s001.zip › ijms-4399494-supplementary.pdf]
